# A pathologically expanded, clonal lineage of IL-21–producing CD4^+^ T cells drives inflammatory neuropathy

**DOI:** 10.1172/JCI178602

**Published:** 2024-06-11

**Authors:** Maryamsadat Seyedsadr, Madison F. Bang, Ethan C. McCarthy, Shirley Zhang, Ho-Chung Chen, Mahnia Mohebbi, Willy Hugo, Jason K. Whitmire, Melissa G. Lechner, Maureen A. Su

**Affiliations:** 1 Department of Microbiology, Immunology, and Molecular Genetics and; 2 Department of Medicine, UCLA David Geffen School of Medicine, Los Angeles, California, USA.; 3 Department of Genetics, UNC Chapel Hill, Chapel Hill, North Carolina, USA.; 4 Department of Pediatrics, UCLA David Geffen School of Medicine, Los Angeles, California, USA.

**Keywords:** Autoimmunity, Immunology, Autoimmune diseases, Cytokines, T cells

## Abstract

Inflammatory neuropathies, which include chronic inflammatory demyelinating polyneuropathy (CIDP) and Guillain Barré syndrome (GBS), result from autoimmune destruction of the PNS and are characterized by progressive weakness and sensory loss. CD4^+^ T cells play a key role in the autoimmune destruction of the PNS. Yet, key properties of pathogenic CD4^+^ T cells remain incompletely understood. Here, we used paired single-cell RNA-Seq (scRNA-Seq) and single-cell T cell receptor–sequencing (scTCR-Seq) of peripheral nerves from an inflammatory neuropathy mouse model to identify IL-21–expressing CD4^+^ T cells that were clonally expanded and multifunctional. These IL-21–expressing CD4^+^ T cells consisted of 2 transcriptionally distinct expanded cell populations, which expressed genes associated with T follicular helper (Tfh) and T peripheral helper (Tph) cell subsets. Remarkably, TCR clonotypes were shared between these 2 IL-21–expressing cell populations, suggesting a common lineage differentiation pathway. Finally, we demonstrated that IL-21 receptor–KO (IL-21R–KO) mice were protected from neuropathy development and had decreased immune infiltration into peripheral nerves. IL-21 signaling upregulated CXCR6, a chemokine receptor that promotes CD4^+^ T cell localization in peripheral nerves. Together, these findings point to IL-21 signaling, Tfh/Tph differentiation, and CXCR6-mediated cellular localization as potential therapeutic targets in inflammatory neuropathies.

## Abstract 2

IL-21–expressing CD4^+^ T cells, as crucial pathogenic immune cell types, characterize mouse models of autoimmune peripheral neuropathies similar to chronic inflammatory demyelinating polyneuropathy and Guillain-Barré syndrome.

## Graphical abstract

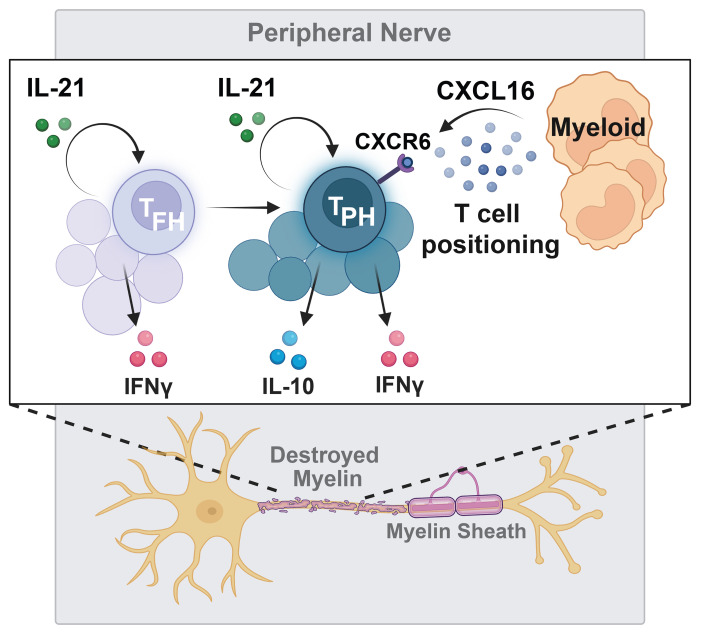

## Introduction

Inflammatory neuropathies, which include chronic inflammatory demyelinating polyneuropathy (CIDP) and Guillain Barré syndrome (GBS), are characterized by debilitating weakness and sensory loss. Hallmarks of these conditions include autoimmune demyelination and immune cell infiltration of peripheral nerves (1, 2). Intravenous immunoglobulin (IVIg) is a mainstay of therapy, but it fails to achieve notable clinical responses in one-third of patients with CIDP p (3), and GBS is associated with a 6.6-fold increase in mortality even with IVIg therapy (4). Moreover, IVIg has broad effects on the immune system that remain incompletely defined (5). Despite the need for more effective, mechanism-based treatments, new therapeutic approaches have not been introduced since the 1990s (6, 7). This contrasts with multiple sclerosis (MS), an autoimmune demyelinating condition of the CNS, for which more than 10 disease-modifying therapies have been FDA approved since 1994 (8).

Progress in developing new immunotherapeutic agents for inflammatory neuropathies has been hampered by a paucity of knowledge regarding fundamental aspects of autoimmune pathogenesis. A major breakthrough in addressing this need has been the recent development of mouse models of inflammatory neuropathies that recapitulate multiple aspects of human disease. For instance, NOD.Aire^GW/^ mice develop spontaneous autoimmune peripheral polyneuropathy (SAPP), which is associated with demyelination and immune cell infiltration in peripheral nerves (9, 10). In this model, autoimmune-prone nonobese diabetic (NOD) mice harbor a partial loss-of-function G228W mutation in the *Aire* (autoimmune regulator) gene, which allows the escape of autoreactive T cells from thymic negative selection. The increased frequency of autoreactive T cells that recognize myelin-specific self-antigens predispose to T cell activation, infiltration into peripheral nerves, and destruction of myelin in peripheral nerves. Importantly, inflammatory neuropathy has also been reported in patients with mutations in the *AIRE* locus (11), highlighting the importance of autoreactive T cells in driving autoimmune peripheral neuropathy across species.

Among T cells, CD4^+^ T cells in particular are critical in the pathogenesis of inflammatory neuropathies. CD4^+^ T cells are increased in peripheral nerves of patients with inflammatory neuropathies (12–14) and SAPP mouse models (10), suggesting a role for CD4^+^ T cells in peripheral nerve myelin destruction. Moreover, CD4^+^ T cells from neuropathic mice are sufficient to transfer SAPP to immunodeficient recipients (9, 10), and a myelin-specific CD4^+^ TCR–transgenic mouse model spontaneously develops autoimmune peripheral neuropathy, suggesting that CD4^+^ T cells are sufficient for the development of autoimmunity (10, 15, 16). Despite these findings that support the importance of CD4^+^ T cells, key properties of pathogenic CD4^+^ T cells in inflammatory neuropathies remain incompletely understood.

Here, we show that, in peripheral nerve infiltrates of neuropathic NOD.Aire^GW/^ mice, terminally differentiated effector CD4^+^ T cells were clonally expanded and expressed IL-21. These IL-21–producing cells could be grouped into 2 transcriptionally distinct populations, which resembled T follicular helper (Tfh) and T peripheral helper (Tph) cells. Notably, TCR clonotypes were shared in these 2 subsets, supporting the idea of a common lineage for these 2 cell populations. Additionally, we demonstrate that IL-21 signaling was required for neuropathy development and that IL-21 upregulated CXCR6, a chemokine that promotes CD4^+^ T cell localization within peripheral nerves. Together, these findings demonstrate a critical role for IL-21 in disease pathogenesis and reveal multiple new molecular targets for the treatment of autoimmune peripheral neuropathies.

## Results

### IL-21–expressing CD4^+^ T cells are pathologically expanded in peripheral nerve autoimmunity.

**Figure 1:**
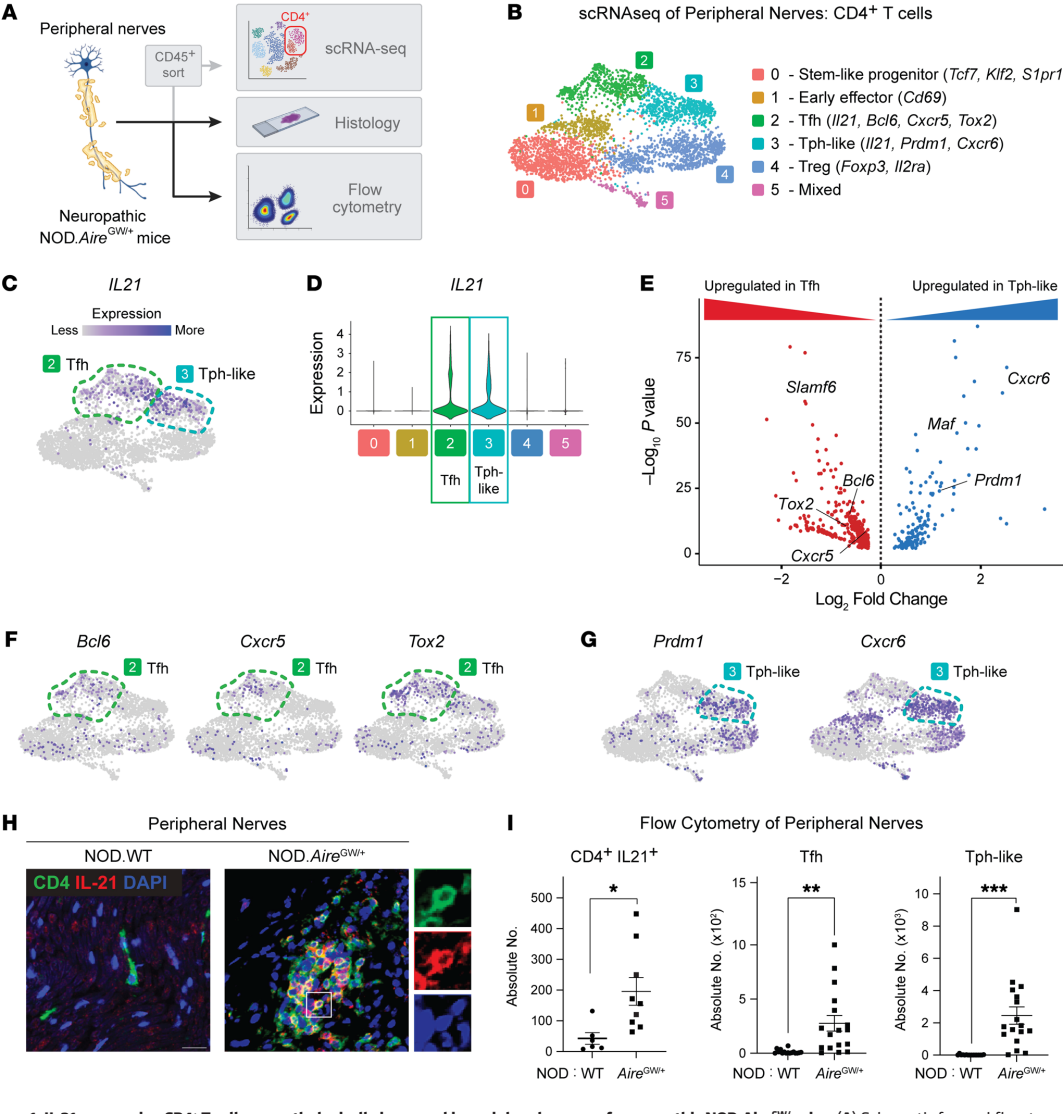
IL-21–expressing CD4^+^ T cells are pathologically increased in peripheral nerves of neuropathic NOD.Aire^GW/^ mice. (**A**) Schematic for workflow to analyze peripheral nerve–infiltrating CD4^+^ T cells in neuropathic NOD.Aire^GW/^ mice. (**B**) UMAP plot of infiltrating CD4^+^ T cells in the sciatic nerves of NOD.Aire^GW/^ mice (*n* = 7). (**C** and **D**) Feature plot (**C**) and violin plot (**D**) of *Il21* expression. (**E**) Volcano plot of DEGs between cells in Tfh versus Tph-like clusters (log_2_ FC > 0.05, *P* < 0.05). (**F** and **G**) Feature plots showing the expression of key transcription factors and chemokines associated with Tfh (*Bcl6, Tox2, Cxcr5*) and Tph (*Prdm1*, *Cxcr6*) cells. (**H**) Microscopy images of immunofluorescence staining for CD4, IL-21, and DAPI in NOD.WT (control) and neuropathic NOD.Aire^GW/^ sciatic nerves. Right panels show the area outlined by the white box, magnified, and split by fluorescence. Scale bar: 20 µm. Original magnification ×20. (**I**) Absolute numbers of the indicated cell types per sciatic nerve, as determined by flow cytometric analysis. CD4^+^ T cells that produced IL-21, Tfh cells (CD4^+^ICOS^+^PD-1^+^CXCR5^+^), and Tph-like cells (CD4^+^ICOS^+^PD-1^+^CXCR5^–^CXCR6^+^) were compared between NOD.WT and neuropathic NOD.Aire^GW/^ sciatic nerves. *P < 0.05, **P < 0.01, and ***P < 0.001, by Mann-Whitney *U* test.

Previous studies have demonstrated infiltrating CD4^+^ T cells in peripheral nerve biopsies from both patients with inflammatory neuropathy (12, 13) and SAPP mouse models (10, 15), supporting an important role for CD4^+^ T cells in the development of PNS autoimmunity. To gain deeper insights into the phenotype of pathogenic CD4^+^ T cells, we analyzed sciatic nerve–infiltrating CD4^+^ T cells from neuropathic NOD.Aire^GW/^ mice (Figure 1A). Single-cell RNA-Seq (scRNA-Seq) analysis of 4,017 CD4^+^ T cells revealed 6 distinct groups (Figure 1B and Supporting Data Values; supplemental material available online with this article; https://doi.org/10.1172/JCI178602DS1). These included clusters differentially expressing stem-like progenitor genes (17–19) (*Tcf7*, *Klf2*, and *S1pr1*; cluster 0*)*; the early lymphocyte activation marker *Cd69* (20, 21) (cluster 1); Treg genes (*Foxp3*, *Il2ra;* cluster 4*)*; and a mix of lineage-associated genes (cluster 5) (Supplemental Figure 1, A and B).

Remarkably, 2 CD4^+^ T cell populations (clusters 2 and 3) significantly upregulated IL-21 (Figure 1, C and D, and Supplemental Figure 1A), a cytokine linked to type 1 diabetes and other autoimmune conditions but not yet to PNS autoimmunity (22, 23). In contrast, IL-21 expression was absent in immune cells found in sciatic nerves of non-neuropathic wild-type NOD (NOD.WT) mice (Supplemental Figure 1, C and D) (24). Thus, the development of autoimmune peripheral neuropathy in NOD.Aire^GW/^ mice was associated with IL-21 upregulation in peripheral nerve CD4^+^ T cells.

Comparison of the two IL-21–producing cell populations (clusters 2 and 3) showed distinct transcriptional profiles, with 560 differentially regulated genes (adjusted *P* < 0.05; log_2_ fold change [FC] > 0.5). Expression of genes (*Bcl6*, *Tox2*, *Cxcr5*, *Slamf6*) associated with Tfh cells (25, 26) was significantly upregulated in cluster 2, whereas expression of genes (*Prdm1* [also known as *Blimp1*], *Cxcr6*) associated with Tph cells (27–29) was significantly upregulated in cluster 3 (Figure 1E). In accordance with this, feature plots showed Tfh-associated genes (*Bcl6*, *Tox2*, *Cxcr5*) expressed by cells in cluster 2 and Tph-associated genes (*Prdm1*, *Cxcr6)* genes expressed by cells in cluster 3 (Figure 1, F and G). Of note, cluster 3 failed to express the subset of Tph-associated chemokines (e.g., *Cxcl13*, *Ccr2*, *Cx3cr1*) described in rheumatoid arthritis and other conditions (27), a finding consistent with previous reports that chemokine expression by Tph cells is context dependent (30). Together, these data suggest that Tfh (cluster 2) and Tph-like (cluster 3) cells are the primary source of IL-21 within the inflamed nerve.

We verified these scRNA-Seq findings using multiple complementary approaches. First, immunofluorescence staining of frozen sciatic nerve sections revealed colocalization of CD4 (green) and IL-21 (red) in neuropathic NOD.Aire^GW/^ sciatic nerves, indicating the production of IL-21 by CD4^+^ T cells in inflamed peripheral nerves. Moreover, CD4 and IL-21 staining was absent in the non-neuropathic controls (NOD.WT) (Figure 1H), which suggests that the neuropathy was associated with increased IL-2–producing CD4^+^ T cells in peripheral nerves. Second, flow cytometric analysis of intracellular IL-21 cytokine staining showed accumulation of IL-21^+^CD4^+^ T cells in NOD.Aire^GW/^ sciatic nerves but not in non-neuropathic NOD.WT control nerves (Supplemental Figure 2A and Figure 1I). Collectively, these data support our scRNA-Seq analysis which showed that IL-21–producing CD4^+^ T cells were pathologically expanded within the sciatic nerves of neuropathic NOD.Aire^GW/^ mice.

Additionally, we performed flow cytometry to quantify Tfh and Tph-like cells in inflamed nerves. Tfh cells are classically identified as CD4^+^ICOS^+^PD-1^+^CXCR5^+^ by flow cytometry, whereas Tph cells lack CXCR5 expression and are therefore identified as CD4^+^ICOS^+^PD-1^+^CXCR5^–^ (25, 26, 30) (Supplemental Figure 2B). Because our scRNA-Seq analysis showed CXCR6 upregulation in the cluster of Tph-like cells (Figure 1, E and G and Supplemental Figure 1A), we additionally incorporated CXCR6 as a marker for Tph-like cells (Supplemental Figure 2B). Consistent with previous reports (27), ICOS expression was higher in Tfh and Tph subsets compared with non-Tfh/Tph cells (Supplemental Figure 2C), and BCL6 transcription factor expression was higher in Tfh cells than in Tph-like cells (Supplemental Figure 2D). Within the immune cell infiltrate of sciatic nerves of neuropathic NOD.Aire^GW/^ mice, we found increased numbers of Tfh and Tph cells compared with non-neuropathic NOD.WT controls (Figure 1I). These findings, together with our scRNA-Seq analyses, revealed pathologic expansion of IL-21–producing Tfh and Tph-like cell populations in inflamed nerves of neuropathic mice.

### IL-21–producing cells in infiltrated peripheral nerves share a common lineage progenitor.

Tfh and Tph cells are reported to share a number of phenotypic features, including IL-21 cytokine expression, presence in inflamed tissue, function in promoting B and T cell activation and maturation, and expression of the cell-surface proteins ICOS and programmed cell death protein 1 (PD-1) (27, 30, 31). These findings suggest a close molecular relationship between these 2cell populations. On the other hand, Tfh and Tph cells are reported to be transcriptionally distinct (27), and we found that, in inflamed peripheral nerves, Tfh and Tph-like cells had divergent gene expression profiles (Figure 1E). Thus, it remains unclear whether Tfh and Tph-like cells are developmentally related, both in inflamed nerves and other contexts.

**Figure 2:**
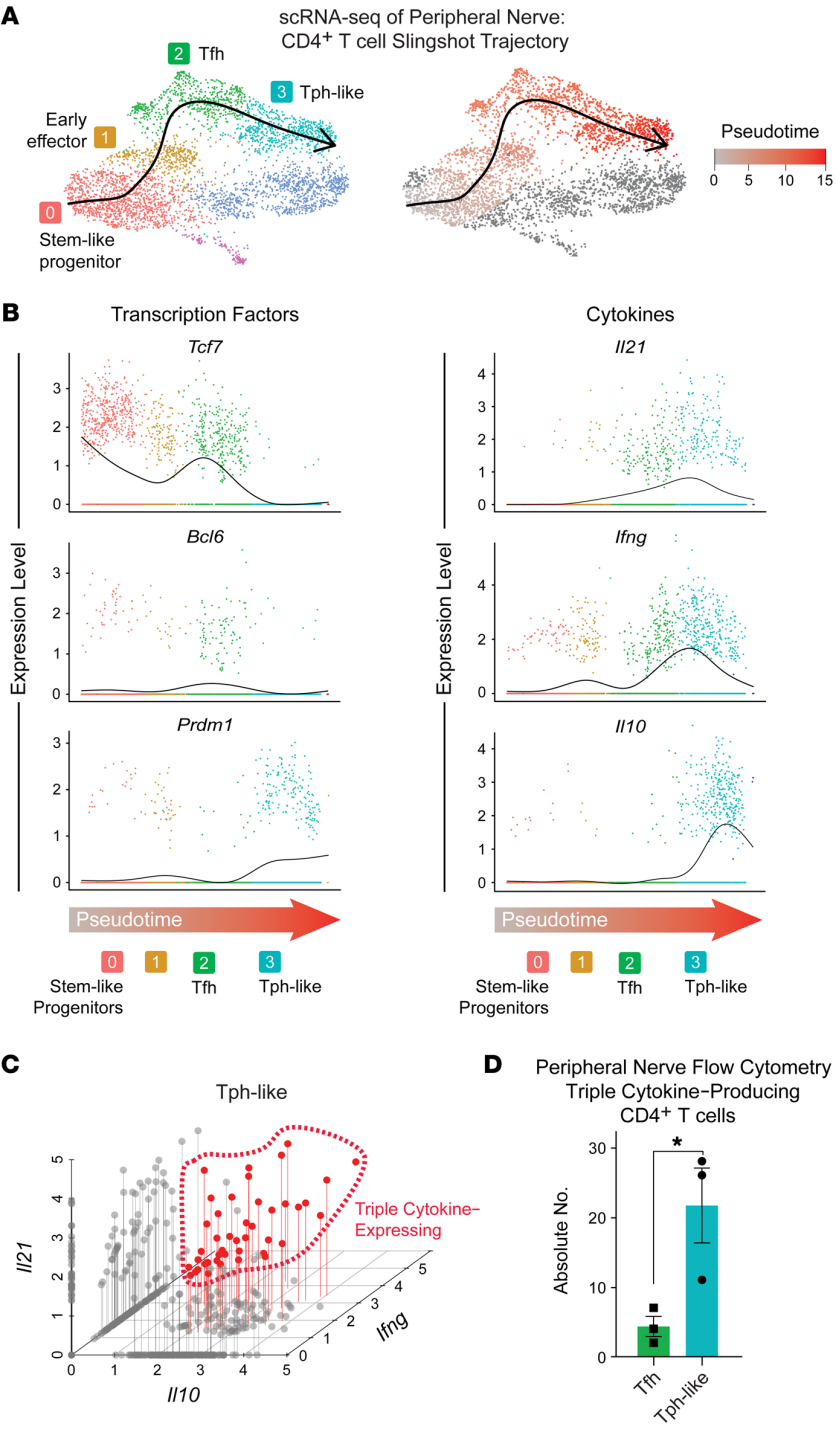
IL-21–producing cells in infiltrated peripheral nerves share a common lineage. (**A**) UMAP plot of CD4^+^ T cells with overlaid Slingshot pseudotime trajectory (left) and with cells color coded chronologically along pseudotime (gray represents the least differentiated and red indicates the most differentiated). (**B**) Expression of key genes along the Slingshot pseudotime trajectory, color coded by the clusters shown in **B** (left). (**C**) *Il21*, *Ifng*, and *Il10* coexpression by single cells within the Tph-like cluster. Triple cytokine–producing cells are circled. (**D**) Flow cytometric analysis of intracellular IL-21, IFN-γ, and Il-10 staining of peripheral nerve Tfh and Tph-like cells from neuropathic NOD.Aire^GW/^peripheral nerves. *P < 0.05, by unpaired 2-tailed *t* test.

To begin to define the ontogeny of these cells, we examined transcriptional transitions of PNS-infiltrating CD4^+^ T cells from neuropathic NOD.Aire^GW/^ mice. Slingshot pseudotime analysis of our scRNA-Seq data revealed 3 trajectories (Supplemental Figure 3A), including 1 trajectory in which CD4^+^ T cells originated from stem-like progenitors (*Tcf7*, *S1pr1*, *Klf2*), progressed through early effectors (*Cd69*) and Tfh cell (*Bcl6*, *Tox2*, *Cxcr5*) states, and terminated with Tph-like cells (*Prdm1*, *Cxcr6*, *Cxcr5^–^*) (Figure 2A). As cells progressed from stem-like progenitors, they decreased the expression of the stem-like progenitor transcription factor *Tcf7*, while upregulating the Tfh-associated transcription factor *Bcl6* and, finally, the Tph-associated transcription factor *Prdm1* (Figure 2B). These data suggest that Tph-like cells in SAPP arose from the Tfh cell population. Moreover, CD4^+^ T cells not only upregulated *Il21* as they progressed along this trajectory, but also *Ifng* and *Il10*. Because IFN-γ and IL-10 have both been identified as disease-promoting cytokines in SAPP (32, 33), these findings suggest that CD4^+^ T cells acquired an autoimmune effector phenotype as they differentiated along this lineage toward Tph-like cells.

The presence of stem-like progenitors and Tfh and Tph-like cells was validated in NOD.Aire^GW/^ peripheral nerves by flow cytometry (Supplemental Figure 3B). We first identified Tfh cells as PD-1^+^CXCR5^+^ among CD4^+^CD62L^–^ cells. We then utilized Ly108 (encoded by *Slamf6*) as a marker of CD4^+^ T cell stem-like progenitors (34). Because Ly108 is also highly expressed by Tfh cells (34), we gated out Tfh cells before identifying Ly108^+^CXCR6^–^ cells as stem-like progenitors. Finally, Tph cells were identified as non-Tfh cells (CXCR5^–^) that were also Ly108^–^CXCR6^+^.

While our data show that expression of IL-21, IFN-γ, and IL-10 was highest for each in cells at the end of the pseudotime trajectory (Figure 2B), whether a single CD4^+^ T cell was capable of transcribing all 3 cytokines is unclear. To assess this, we correlated the expression of IL-21, IFN-γ, and IL-10 within single cells. Using our scRNA-seq data set to query cytokine expression in a single Tph-like cell, we found that 26% of cells expressed 2 of the 3 cytokines and 7% expressed all 3 (Figure 2C). The multifunctionality of CD4^+^ T cells was confirmed by intracellular cytokine staining and flow cytometric analysis (Supplemental Figure 3C and Figure 2D), which demonstrated triple cytokine expression by a subset of Tph-like cells in neuropathic NOD.Aire^GW/^ sciatic nerves. Thus, simultaneous expression of IL-21, IFN-γ, and IL-10 was observed in a subset of Tph-like cells in inflamed nerves of SAPP mice. This finding mirrors the coexpression of IL-21, IFN-γ, and IL-10 by pathogenic Tph cells in rheumatoid arthritis (27) and Tph-like cells in kidney injury (35).

### Multifunctional Tph-like cells are clonally expanded in peripheral nerves.

**Figure 3:**
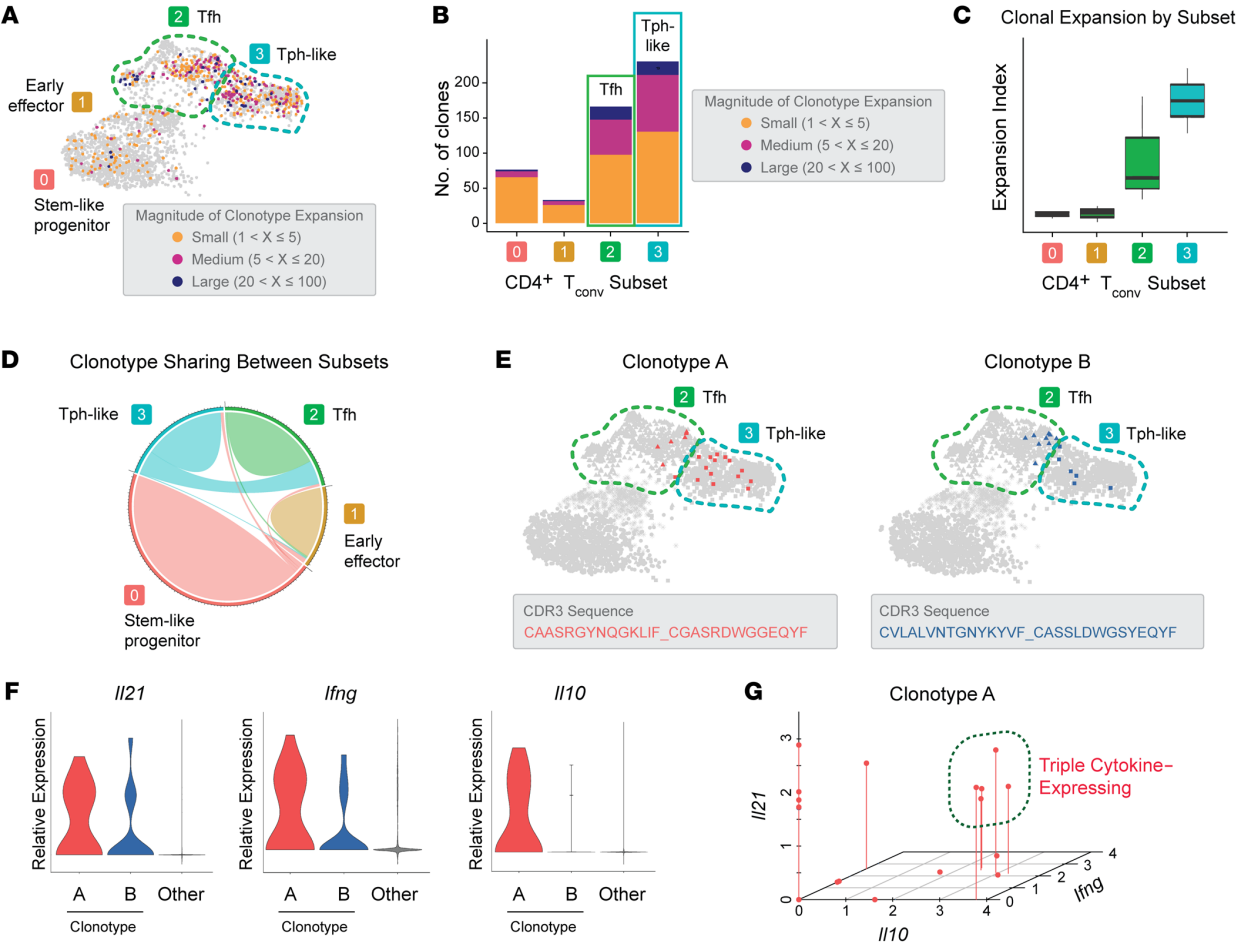
Tph-like cells in infiltrated peripheral nerves are clonally expanded and express IL-21, IFN-γ, and IL-10. (**A**) UMAP of peripheral nerve–infiltrating CD4^+^ Tconv cells with projection of expanded clonotypes. The magnitude of expansion is grouped as small, medium, and large as indicated, with individual cells color coded according to expansion magnitude. (**B**) Numbers of clonally expanded Tconv cells, grouped by cluster. The degree of expansion is indicated by color. (**C**) Clonal expansion levels of Tconv clusters quantified by STARTRAC. (**D**) Chord diagram of clonotype interconnections between clusters. The greatest sharing is seen between Tfh and Tph-like clusters (green). (**E**) Visualization of the 2 expanded clonotypes (clonotypes A and B) by their projection to the UMAP of CD4^+^ Tconv cells. CDR3 sequences for these 2 clonotypes are indicated. (**F**) Violin plots of cytokine expression levels in clonotypes A and B compared with all other cells. (**G**) Correlation plot showing the levels of coexpression of *Ifng*, *Il10*, and *Il21* cytokines by individual cells from clonotype A. Cells expressing all 3 cytokines are circled.

During an autoimmune response, self-reactive T cells undergo clonal expansion with T cell receptor (TCR) self-antigen recognition and subsequent activation. Given the extremely low probability that somatic recombination at the TCR locus will result in the exact V(D)J rearrangement more than once, TCR sequences can be used as unique identifiers of T cell clones (36). To query the clonality of PNS-infiltrating CD4^+^ T cells in neuropathic mice, we analyzed data from paired, single-cell TCR-Seq (scTCR-Seq) and scRNA-Seq of 4 NOD.Aire^GW/^ sciatic nerve samples. The Treg and mixed clusters were removed from this analysis in order to focus on conventional T (Tconv) cells. Using the total number of cells expressing each TCR sequence, we categorized each clonotype expansion as small (1< × ≤5), medium (5< × ≤20), or large (× >20). Most cells associated with medium and large clonal expansions mapped to the Tfh and Tph-like clusters (Figure 3, A and B). Clonality was also measured using the Shannon entropy-based STARTRAC clonality index (37, 38), which demonstrated the highest index scores in Tfh and Tph-like cell populations (Figure 3C). Thus, the greatest degree of clonal expansion occurred in the Tfh and Tph-like groups.

Because of the low likelihood that 2 identical TCR sequences would arise independently in the same mouse, clonal sharing among cells with distinct phenotypes would suggest development from a common progenitor (36). Visualization by chord diagram revealed that the majority of clonal sharing occurred between Tfh and Tph-like clusters (Figure 3D) (39, 40). Mapping of individual cells belonging to specific highly expanded clonotypes (i.e., clonotype A and clonotype B) also demonstrated sharing between Tfh and Tph-like cells (Figure 3E). For instance, cells from clonotype A mapped to both Tfh and Tph-like clusters. This evidence of clonotype sharing between cells in the Tfh and Tph-like clusters suggests that Tfh and Tph-like cells originated from a shared precursor. These cells then proliferated in response to TCR activation and differentiated into distinct subsets.

We next examined the link between cytokine expression and clonal expansion (Figure 3F). Notably, we found that *Il21* was highly expressed by cells associated with clonotypes A and B compared with all other cells. Similarly, *Ifng* was also highly expressed by clonotypes A and B. However, we found that *IL10* was highly expressed by clonotype A but not by clonotype B. Interestingly, analysis of gene expression within single cells revealed a subset of cells associated with clonotype A that simultaneously expressed all 3 cytokines (Figure 3G). Together, these data identify clonally expanded cells that traversed Tfh and Tph-like clusters and were capable of simultaneously expressing IL-21 and the pathogenic cytokines IFN-γ and IL-10.

We utilized 79-6, a pharmacological inhibitor of the Tfh transcription factor BCL6, to empirically determine whether Tfh cells differentiate into Tph cells (Supplemental Figure 4A). In an adoptive transfer model of autoimmune neuropathy, we found that 79-6 treatment was accompanied by a decrease in the frequency of nerve-infiltrating Tph (CD4^+^ICOS^+^CXCR5^–^PD-1^+^CXCR6^+^) cells compared with vehicle treatment (Supplemental Figure 4B). In addition, treatment with 79-6 significantly reduced neuropathy incidence and improved sciatic nerve conduction parameters (Supplemental Figure 4, C and D). These findings suggest that inhibiting Tfh-associated BCL6 can ameliorate neuropathy by reducing the Tph cell population.

### IL-21 signaling is essential for the development of autoimmune peripheral neuropathy.

**Figure 4:**
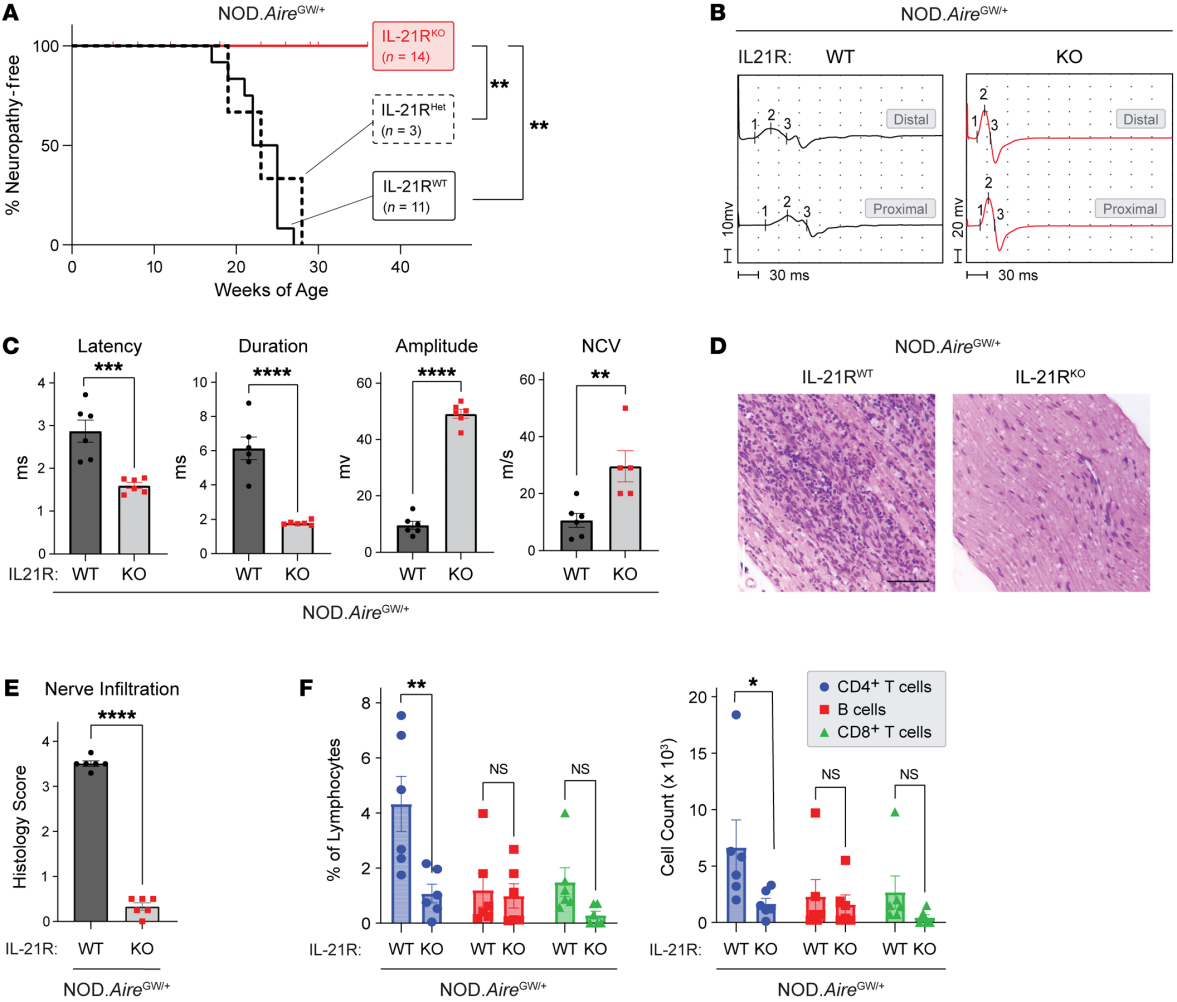
IL-21R signaling is required for neuropathy development in NOD.Aire^GW/^ mice. (**A**) Neuropathy-free incidence curve for female NOD.Aire^GW/^ IL-21R^KO^ versus NOD.Aire^GW/^ IL-21R^Het^ versus NOD.Aire^GW/^ IL-21R^WT^ mice. **P < 0.01, by Mantel-Cox test. (**B** and **C**) Representative distal and proximal compound muscle action potentials from IL-21R–sufficient versus IL-21R–deficient NOD.Aire^GW/^ sciatic nerves. The latency, duration, amplitude, and NCV were quantified and compared between groups (*n* = 6). **P < 0.01, ***P < 0.001, and ****P < 0.0001, by unpaired 2-tailed *t* test. (**D** and **E**) Immune cell infiltration scores in the forelimb nerves as assessed by H&E staining and compared between groups (*n* = 6). ****P < 0.0001, by unpaired 2-tailed *t* test. Scale bar: 200 μm. (**F**) The frequency and number of CD4^+^ T cells, B lymphocytes (CD4^–^CD8^–^B220^+^), and CD8^+^ T cells were compared between groups in the peripheral nerves (*n* = 6). *P < 0.05 and **P < 0.01, by 2-way ANOVA with Bonferroni’s multiple correction.

Although IFN-γ and IL-10 have been implicated in SAPP pathogenesis (32, 33), the role of IL-21 remains unclear. Upregulation of IL-21 in infiltrating CD4^+^ T cells and its expression by clonally expanded T cells suggest a critical role for IL-21 signaling in PNS autoimmunity development. To investigate this, we generated female NOD.Aire^GW/^ mice with 1 or 2 copies of loss-of-function mutations in the IL-21 receptor (IL-21R). Female NOD.Aire^GW/^ mice with a heterozygous mutation in the IL-21R (NOD.Aire^GW/^ IL-21R^Het^ mice) developed neuropathy with the same onset and incidence as NOD.Aire^GW/^ mice sufficient for the IL-21R (NOD.Aire^GW/^ IL-21R^WT^) (Figure 4A). In contrast, female NOD.Aire^GW/^ mice with homozygous mutations in the IL-21R (NOD.Aire^GW/^ IL-21^KO^ mice) were protected against SAPP (Figure 4A). This protective effect of IL-21R deficiency was not sex dependent, since IL-21R deficiency was also protective in male NOD.Aire^GW/^ mice (Supplemental Figure 5A).

We have previously reported that female NOD.Aire^GW/^ mice show evidence of demyelination on motor nerve electrophysiology (32). In comparison, compound muscle action potentials from IL-21R–deficient NOD.Aire^GW/^ mice showed improvement in multiple parameters, including reduced latency and duration and increased amplitude and nerve conduction velocity (NCV) (Figure 4, B and C). Additionally, histological analysis revealed significantly reduced peripheral nerve infiltration in NOD.Aire^GW/^ IL-21R^KO^ mice compared with NOD.Aire^GW/^ IL-21R^WT^ mice (Figure 4, D and E). Thus, our findings indicate a critical role for IL-21 signaling in the development of SAPP. In addition to genetically ablation of IL-21 signaling, we assessed the efficacy of IL-21R monoclonal antibody in an adoptive transfer model. The experimental group treated with anti–IL-21R antibody showed a decreasing trend in the incidence of neuropathy, electromyography (EMG) abnormalities, and CD4^+^ T cell infiltration (Supplemental Figure 5, B–D).

IL-21 has broad cellular targets, since the IL-21R is expressed by various immune cell types (e.g., CD4^+^ T cells, CD8^+^ T cells, B cells) (41). Flow cytometric analysis of peripheral nerve immune infiltrate indicated lower numbers of CD4^+^ T cells, with no significant change in CD8^+^ T cells or B220^+^ B cells (Figure 4F). In “mix-and-match” adoptive transfer experiments, in which CD4^+^ and CD8^+^ T cells were either from IL-21R–sufficient or IL-21R-deficient NOD.Aire^GW/^ mice, a modest delay in the development of neuropathy was noted when CD4^+^ T cells were IL-21R deficient. This finding suggests that blocking IL-21R signaling in CD4^+^ T cells was sufficient to delay neuropathy (Supplemental Figure 6A).

Of note, the absolute number of DCs and macrophages was also decreased in the peripheral nerves of Aire^GW/^ IL-21R^KO^ mice in comparison with Aire^GW/^ IL-21R^WT^ mice (Supplemental Figure 6B). Thus, it is possible that IL-21 signaling on DCs and macrophages may also play a role in promoting neuropathy. Finally, peripheral nerve–infiltrating CD4^+^ T cells from IL-21R–deficient mice demonstrated a decrease in IL-21–, IFN-γ–, and IL-10–producing CD4^+^ T cells (Supplemental Figure 6C), suggesting that IL-21 from CD4^+^ T cells signaled in an autocrine manner to increase the numbers of cytokine-producing CD4^+^ T cells in inflamed peripheral nerves. However, it remains unclear if IL-21 regulated signals important for positioning pathogenic CD4^+^ T cells within inflamed peripheral nerves.

### CXCR6 upregulation in CD4^+^ T cells is IL-21 dependent.

**Figure 5:**
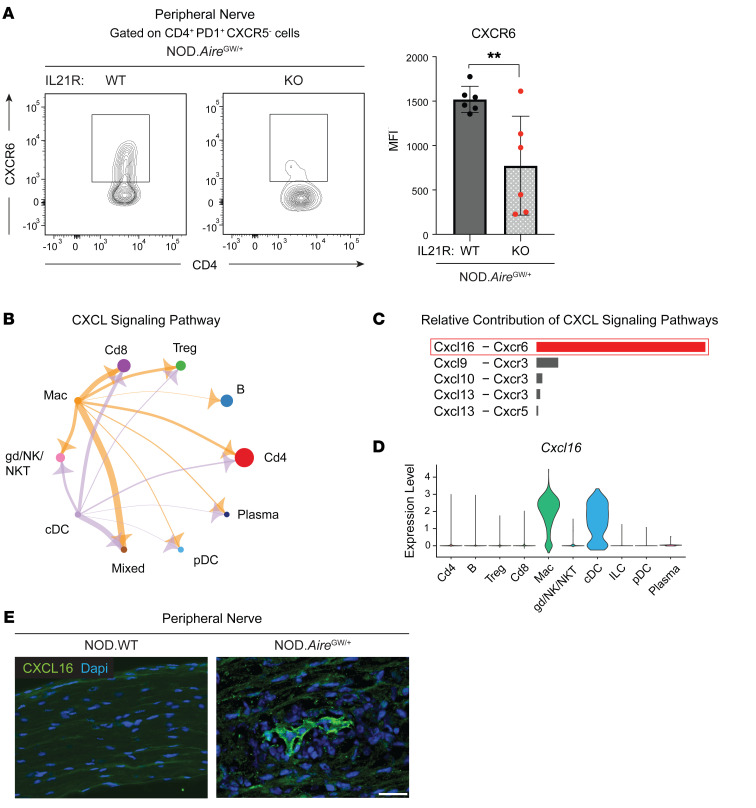
IL-21–mediated CXCR6 upregulation is required for the recruitment of CD4 cells to the peripheral nerves. (**A**) Flow cytometric analysis of CXCR6 expression on CD4^+^ Tph cells from IL-21R–sufficient versus IL-21R–deficient NOD.Aire^GW/^ sciatic nerves (*n* = 6). **P < 0.01, by 2-tailed unpaired *t* test. (**B**) CellChat analysis of ligand-receptor interactions shows upregulation of the CXCL signaling pathway. (**C**) Relative contribution of chemokine ligands and corresponding receptors in the sciatic nerves of NOD.Aire^GW/^ mice. (**D**) Expression of CXCL16 in infiltrated immune cell subsets. Highest expression was seen in macrophages (Mac) and cDCs. (**E**) Immunostaining for CXCL16 in peripheral nerves of NOD.WT and NOD.Aire^GW/^ neuropathic mice. Scale bar: 20 μm.

Our initial scRNA-Seq analysis revealed that the chemokine receptor CXCR6 was upregulated in Tph-like cells in peripheral nerves of neuropathic NOD.Aire^GW/^ mice (Figure 1G and Supplemental Figure 1A). Notably, flow cytometric analysis of Tph-like (CD4^+^PD-1^+^CXCR5^–^) cells in peripheral nerves revealed significantly lower CXCR6 MFI in IL-21R–deficient NOD.Aire^GW/^ mice compared with IL-21R–sufficient controls (Figure 5A). These in vivo findings are in accord with previously published microarray data, which show that IL-21 stimulation in vitro upregulates CD4^+^ T cell expression of *Cxcr6* (Supplemental Figure 6) (42). Thus, CXCR6 expression by CD4^+^ T cells was IL-21 dependent.

To identify molecular mechanisms governing T cell positioning within inflamed peripheral nerves, we analyzed a previously published scRNA-Seq data set of NOD.Aire^GW/^ nerve-infiltrating immune cells (Gene Expression Omnibus [GEO] GSE180498). Using the CellChat R package to characterize ligand-receptor interactions, we identified upregulation of the “CXCL signaling pathway” (Figure 5B), with prominent interactions between myeloid cells and T cells. Of these interactions, CXCL16-CXCR6 pairs were the most upregulated of the CXCL signaling pathways (Figure 5C). CXCL16 is the only known ligand for CXCR6, and CXCL16-CXCR6 interactions have been reported to play an important role in positioning T cells in tumors and other tissues (29, 43, 44). Whether CXCL16-CXCR6 interactions play a role in positioning pathogenic T cells in inflamed peripheral nerves, however, is unknown.

In support of an important role for CXCL16-CXCR6 interactions, *Cxcl16* and *Cxcr6* expression levels were higher in infiltrating immune cells of neuropathic NOD.Aire^GW/^ nerves, compared with non-neuropathic NOD.WT controls (Supplemental Figure 7A). *Cxcl16* was highly expressed by macrophages and conventional DCs (cDCs) in neuropathic NOD.Aire^GW/^ nerves, whereas *Cxcr6* was expressed by lymphocytes (Figure 5D and Supplemental Figure 7A). In vitro, CXCL16 expression was upregulated in bone marrow–derived macrophages (BMDMs) and RAW 264.7 cells in response to IFN-γ (Supplemental Figure 7, B and C). Immunofluorescence staining of peripheral nerves from NOD.Aire^GW/^ mice confirmed CXCL16 expression, which was absent in non-neuropathic NOD.WT controls (Figure 5E). Together, these data led us to hypothesize that CXCL16-CXCR6 interactions are important in positioning CD4^+^ T cells within inflamed peripheral nerves and that downregulation of *Cxcr6* with IL-21R deficiency prevents CD4^+^ T cell accumulation in inflamed peripheral nerves.

### IL-21–dependent CXCR6 assists autoreactive CD4^+^ T cell localization to the peripheral nerve.

**Figure 6:**
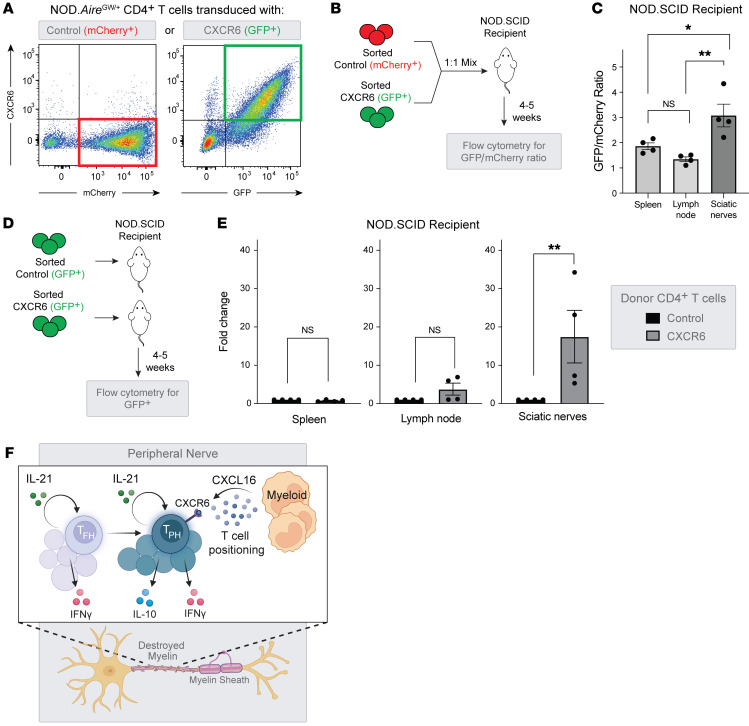
CXCR6 facilitates CD4^+^ T cell localization to inflamed peripheral nerves. (**A**) Flow cytometric plots of CD4^+^ cells from NOD.Aire^GW/^ mice transduced with empty vector (mCherry) or CXCR6-GFP vector. (**B**) Experimental design for adoptive transfer of CXCR6 and control vector–transduced CD4^+^ cells, mixed 1:1 prior to transfer into NOD.SCID recipients. (**C**) Quantification of the GFP (CXCR6 vector) to mCherry (empty vector) ratio in spleens, lymph nodes, and sciatic nerves of recipient SCID mice (*n* = 4). *P < 0.05 and **P < 0.01, by paired 2-tailed *t* test. (**D**) Experimental design for adoptive transfer of CXCR6 or control vector–transduced CD4^+^ cells into NOD.SCID recipients. (**E**) The FC difference in sciatic nerves, lymph nodes, and spleens calculated by normalizing the number of cells to that of the control-transduced group (*n* = 5). **P < 0.01, by paired 2-tailed *t* test. (**F**) Model for how pathogenic CD4^+^ T cells accumulate in inflamed peripheral nerves. Autocrine IL-21 signaling leads to CXCR6 upregulation in Tph-like cells, which allows CXCR6 to interact with CXCL16 expressed by myeloid cells in inflamed peripheral nerves.

To test the role of CXCL16-CXCR6 interactions, we transduced neuropathic NOD.Aire^GW/^ splenic CD4^+^ T cells with a viral vector coexpressing CXCR6 and a GFP reporter (Figure 6A). As a negative control, cells were transduced with an empty vector expressing only an mCherry reporter. To determine the in vivo capacity of CXCR6-overexpressing CD4^+^ T cells to localize to peripheral nerves, CXCR6-overexpressing and control cells were sorted according to reporter gene expression and cotransferred as a 1:1 mix to the same NOD.SCID recipient (Figure 6B). This allowed for assessment of both CD4^+^ T cell groups within the same host environment. Cell distribution was assessed by flow cytometry 4–5 weeks after adoptive transfer, prior to the onset of clinical neuropathy. Although the relative numbers of CXCR6-overexpressing cells versus control cells (GFP/mCherry ratio) were approximately equivalent in the spleen and lymph nodes, the relative numbers of CXCR6-overexpressing cells were higher in the peripheral nerves (Figure 6C). In parallel, we also transferred sorted CXCR6-overexpressing and control cells into separate NOD.SCID hosts (Figure 6D). In this experimental setup, the absolute number of CXCR6-overexpressing CD4^+^ T cells was also increased in peripheral nerves compared with control CD4^+^ T cells (Figure 6E). In contrast, we observed no differences in CD4^+^ T cell counts in the spleen or lymph nodes. Together, these data support a model in which IL-21–dependent expression of CXCR6 in CD4^+^ T cells promotes their localization within the inflamed tissues of the PNS (Figure 6F).

## Discussion

Understanding the autoimmune pathogenesis of inflammatory neuropathies has been greatly facilitated by the development of SAPP mouse models. These models, along with clinical observations in patients, have demonstrated a critical role for CD4^+^ T cells in the development of autoimmune peripheral neuropathy. Nevertheless, much remains unknown about effector mechanisms, ontogeny, and peripheral nerve localization of PNS-reactive CD4^+^ T cells. In this study, we demonstrate that IL-21 production is a hallmark of pathologically expanded, clonally related CD4^+^ T cells in infiltrated peripheral nerves. Genetic IL-21R deficiency completely protected against neuropathy development, demonstrating that IL-21 signaling was required for autoimmune pathogenesis. Finally, we show that IL-21 upregulated the chemokine receptor CXCR6 in CD4^+^ T cells, suggesting a role for IL-21 in CD4^+^ T cell positioning. These findings point to IL-21/IL-21R and CXCR6/CXCL16 as promising targets for therapies in inflammatory neuropathies.

Clonal expansion of CD4^+^ T cells in the NOD.Aire^GW/^ model of autoimmune peripheral neuropathy may reflect escape of PNS-reactive clones from the Aire-deficient thymus. Escape of autoreactive T cell clones from central tolerance mechanisms has previously been reported in Aire-deficient mouse models (45), and further clonal expansion may occur with antigen encounter and cytokine stimulation. Most patients with inflammatory neuropathies, however, do not have a defect in the *Aire* gene. Nevertheless, this clonal expansion of CD4^+^ T cells in NOD.Aire^GW/^ mice mirrors the CD4^+^ T cell clonal expansion associated with human GBS (46), suggesting that clonal expansion is a hallmark of inflammatory neuropathies across species.

The IL-21R is expressed by various immune cell types, which implies that IL-21 has broad cellular targets (41). Previous studies have highlighted the role of IL-21 in CD8^+^ T and B cell activation and differentiation (27). However, the frequency and absolute numbers of CD8^+^ T cells and B220^+^ B cells were unchanged in peripheral nerves of IL-21R–deficient NOD.Aire^GW/^ mice. Instead, our data showed lower numbers of CD4^+^ T cells, suggesting that IL-21 functioned in an autocrine fashion to increase CD4^+^ T cells within peripheral nerve infiltrate. Of note, we have previously reported a pathogenic role for IFN-γ– and IL-10–producing CD4^+^ T cells in SAPP (32, 33), and IL-21R deficiency resulted in a substantial decrease in these cells within peripheral nerves. Additionally, lower numbers of macrophages and DCs were also seen, suggesting a potential role for IL-21 signaling in these myeloid cell types. Finally, we show that IL-21 signaling functioned to upregulate CD4^+^ T cell expression of the chemokine CXCR6, suggesting a potential role for CXCR6 in pathogenic CD4^+^ T cell accumulation in peripheral nerves.

CXCR6-expressing T cells have been well studied in anticancer immunity, in which CXCR6 is used as a marker of resident memory T cells (43). Within tumors, CXCR6 positions T cells next to perivascular DCs that express the CXCR6 ligand CXCL16 (47). At the same time, CXCR6-expressing T cells are enriched in inflamed tissues of patients with psoriasis and inflammatory arthritis (48), suggesting a pathogenic role for CXCR6 in these autoimmune diseases. Indeed, genetic CXCR6 deficiency is protective in mouse models of arthritis (49), and antibody-mediated blockade of CXCR6 or CXCL16 ameliorates disease in a mouse model of MS (50, 51). Together, these findings demonstrate a critical role for CXCR6-CXCL16 interactions in these autoimmune conditions. Our data suggest that CXCR6-CXCL16 interactions were also critical in PNS autoimmunity. scRNA-Seq analysis of infiltrated peripheral nerves showed accumulation of CXCR6-expressing CD4^+^ T cells and demonstrated that CXCR6 overexpression increased the accumulation of CD4^+^ T cells within nerves. Moreover, we found that high levels of CXCL16 were expressed by macrophages and DCs in peripheral nerve infiltrates, suggesting that CXCR6-CXCL16 interactions promoted the localization of pathogenic CD4^+^ T cells to peripheral nerves.

Within infiltrated peripheral nerves, we identified CD4^+^ T cells associated with an expanded clonotype that were capable of expressing multiple pathogenic cytokines (IL-21, IFN-γ, and IL-10). This same set of cytokines were also expressed by senescence-associated T cells (SATs), a CD153-expressing CD4^+^ T cell population associated with aging and inflammation (35, 52, 53). SATs have been proposed to underlie the increased risk of autoimmunity, since anti-CD153–mediated depletion of SATs ameliorated disease in a mouse model of lupus (52). Whether multifunctional Tph-like cells in inflamed peripheral nerves also express cell senescence features and accumulate with age, however, remains to be explored. However, it is intriguing that the incidence of inflammatory neuropathies increases with advancing age, with the peak age of onset between 70 and 79 years of age for patients with CIDP and above 60 years of age for those with GBS (54, 55). These findings suggest that age is a predisposing factor in the development of inflammatory neuropathies.

Collectively, our findings reveal a number of potential therapeutic targets for inflammatory neuropathies. First, therapies targeting IL-21/IL-21R are under development for type 1 diabetes, rheumatoid arthritis, and psoriasis (23), and our findings suggest that therapies blocking IL-21 or IL-21 signaling may be effective as a therapeutic target for inflammatory neuropathies. Second, therapies that target immune cell localization have been effective in inflammatory colitis and other immune-mediated diseases, and our data suggest that blocking CXCR6-CXCL16 receptor-ligand interactions may be efficacious for mitigating T cell localization to the peripheral nerves. Finally, our finding in this study that the most differentiated, clonally expanded CD4^+^ T cells could be triple cytokine producers suggests that therapies that target multiple cytokine signaling pathways, such as the use of JAK/STAT inhibitors, may be a therapeutic strategy for inflammatory neuropathies.

## Methods

### Sex as a biological variable.

We have previously reported that neuropathy age of onset and incidence are higher in female NOD.Aire^GW/^ mice compared with male mice (32). Here, we used both female and male NOD.Aire^GW/^ IL-21R^KO^ mice to show that IL-21 signaling was required for neuropathy in both sexes (Figure 4A and Supplemental Figure 5A). We subsequently focused on female mice in other studies, given the earlier onset and higher incidence in NOD.Aire^GW/^ females.

### Mice.

NOD.Aire^GW/^ mice (9) and NOD.Cg-Prkdc^scid^/J (NODSCID, JAX 001303) mice were housed in a specific pathogen–free (SPF) barrier facility at UCLA. NOD.Aire^GW/^IL-21R^KO^ mice were produced by crossing NOD.129(Cg)-IL-21R^tm1Wjl^/DcrMmjax (NOD.IL-21RKO, JAX 050918) mice with NOD.Aire^GW/^ mice. In all experiments, female mice were used.

### Neuropathy assessment and nerve conduction studies.

Neuropathy was determined as previously described (56). EMG was performed using a TECA Synergy N2 EMG machine as previously described (57). Compound muscle action potentials (CMAPs) were recorded following the stimulation of the sciatic nerve for 0.1 ms duration at 1 Hz frequency and 20 mA intensity stimulus, with the low-pass filter set to 20 Hz and the high-pass filter to 10 kHz.

### Histology and immunostaining.

The forelimb nerves were dissected for histology and immunostaining. Paraffin-embedded nerve samples were stained with H&E and used for semiquantitative immune cell infiltrate scoring on a scale from 0 to 4 as previously described (10, 32). Three to 4 nonoverlapping microscopic fields were evaluated per nerve. For immunostaining of frozen nerve sections, tissues were fixed in 4% paraformaldehyde overnight and cryopreserved with 30% sucrose for 1–2 days. Nerve samples were embedded in OCT medium (Thermo Fisher Scientific). The frozen blocks were prepared by placing the embedding molds in ethanol cooled by dry ice. Sections (10 μm) were cut from the nerves and collected on Superfrost/Plus slides (Thermo Fisher Scientific). For immunostaining, slides were incubated with primary antibodies overnight at 4°C; the next day, the slides were washed, followed by application of secondary antibodies for 1 hour. The slides were mounted with Fluoromount-G with DAPI (Invitrogen, Thermo Fisher Scientific, 00-4959-52). The list of the antibodies used for immunofluorescence staining can be found in Supplemental Table 1. Images were acquired using a ZEISS Axiocam 208 or ZEISS Axiocam Observer and processed using ImageJ/Fiji software.

### Flow cytometric analysis.

Following cardiac perfusion with PBS, single cells were isolated from the spleen, lymph nodes (2 brachial and 2 inguinal), and sciatic nerves as previously described (56). Briefly, chopped sciatic nerves were digested with 1 mg/mL collagenase IV and passed through a 20 gauge needle. Splenic, lymph node, and digested sciatic nerve samples were passed through 40 μm filters and washed with PBS, yielding single-cell suspensions.

For intracellular cytokine staining, cells were stimulated with PMA (50 ng/mL), ionomycin (1 μg/mL), Brefeldin A (1×), and monensin (1×) for 4 hours. Cells were stained with antibodies against cell-surface proteins and then fixed with Fix & Perm Medium A (Invitrogen, Thermo Fisher Scientific, GAS001S100) followed by permeabilization with Fix & Perm Medium B (Invitrogen, GAS002S100) for cytokine staining. The antibodies used for flow cytometry are listed in Supplemental Table 1. The BD Fortessa Cell Analyzer or Attune NxT Flow Cytometer was used to perform flow cytometry. The flow cytometric data were analyzed using FlowJo software, version 10.

### Cell sorting.

Cells were sorted using a BD FACSAria Cell Sorter at the UCLA BSCRC Flow Cytometry Core as well as a mouse CD4 isolation kit (Miltenyi Biotec, 130-104-454) or a mouse CD8 isolation kit (Miltenyi Biotec, 130-104-075).

### CXCR6 virus preparation and transduction.

The CXCR6-expressing plasmid was generated by subcloning a *Cxcr6* gene block amplified from the ORF (GenScript) into an MSCV-IRES-GFP backbone (Addgene no. 20672). Retrovirus was produced by cotransfecting Phoenix-ECO (American Type Culture Collection [ATCC] CRL-3214) with the transfer plasmids MSCV-CXCR6-IRES-GFP or pMSCV-IRES-mCherry (Addgene no. 52114) and pCL-Eco (Addgene no. 12371) using TransIT-293 transfection reagent (Mirus Bio, catalog 2705). Media were changed 16 hours after transfection. Retrovirus was collected over the 2 following days, and supernatant was stored at –80°C for transduction. Primary murine CD4^+^ T cells were stimulated overnight with plate-bound anti-CD3/anti-CD28 beads before transduction. Transduction was performed on days 1 and 2 by spinoculation at 2,000*g* for 90 minutes at 32^o^C using low acceleration and minimal deceleration. Transduced cells were incubated for 4 days with plate-bound anti-CD3/anti-CD28 in media supplemented with human IL-2 (0.0344 units/mL, Peprotech, no. 200-02). Transduction efficiency was assessed for each experiment by flow cytometry.

### Bone marrow–derived macrophages and RAW cell cultures.

BMDMs were generated using bone marrow collected from a NOD/ShiLtJ (NOD.WT) mouse. Following dissection, the femur and tibia were cut in half and centrifuged to collect marrow. Myeloid bone marrow progenitor cells were cultured for 5 days in complete medium containing 20% L-929 conditioned medium. BMDMs were reseeded and treated with either 215.9 μM DMSO (vehicle), 215.9 μM DMSO and 40 ng/mL recombinant murine IFN-γ, or 10 μM ruxolitnib (Medchem Express, HY-50856). RAW cells were used at the sixth passage. Cells were left untreated or treated with murine IFN-γ for 8.5 hours.

### Quantitative PCR.

RNA extraction was performed using the Quick-RNA Microprep Kit (Zymo Research, product no. R1051) according to the manufacturer’s instructions. cDNA was prepared using the High-Capacity cDNA Reverse Transcription Kit (Applied Biosystems, product no. 4374966), and subsequent quantitative PCR (qPCR) was performed in triplicate using the TaqMan platform (Cxcl16 TaqMan assay ID: Mm00469712_m1, Actb TaqMan assay ID: Mm02619580_m1).

### 79-6 and anti–IL-21R treatment.

The BCL6 inhibitor 79-6 (MilliporeSigma, 197345) was injected i.p (50 mg/kg/d) for 10 days in the following injection solution: 10% DMSO, 40% PEG300, 5% Tween 80, and 45% saline. Anti IL-21R (Bio X Cell, BE0258) was injected i.p with either 500 μg twice per weeks for 2 weeks; 200 μg weekly for 3 weeks; or a 2,500 μg loading dose followed by 500 μg twice per week. The data shown were combined from these experiments.

### scRNA-Seq and analysis.

scRNA-Seq was performed for CD45-isolated cells from the sciatic nerves of 4 neuropathic NOD.Aire^GW/^ mice. We performed 10X Genomics Single Cell Immune Profiling [V(D)J 5′ Gene Expression] at the UCLA Technology Center for Genomics and Bioinformatics (TCGB) core. Analysis was performed using the Seurat package (4.3.0) in R Studio (version 4.2.3, Shortstop Beagle). Raw sequences were processed and mapped to the reference genome (mm10) using CellRanger software (10X Genomics). Outputs of the CellRanger pipeline were read into R Studio with the Read10X function, generating single-cell-level transcript counts of each gene. Cells with a count of fewer than 300 RNAs, more than 5,000 features, and more than 20% mitochondrial genes were excluded from the analysis. Data were normalized and log transformed with the NormalizeData function, and variable features were identified using the FindVariableFeatures function. To maximize cell numbers, this data set was integrated with a previously published data set from 3 neuropathic NOD.Aire^GW/^ mice (GEO GSE180498) (58). Integration anchors were identified among the Seurat object inputs. A single integrated analysis was performed on all *Cd4*-expressing cells. The standard workflow for visualizing and clustering occurred; data were scaled with ScaleData, and linear dimensional reduction (RunPCA) was performed. Cell clusters were determined by the FindClusters function and visualized by uniform manifold approximation and projection (UMAP). Cell types were identified by expression of canonical markers described in existing literature and within the Immgen RNASeq Skyline database (59). Differentially expressed genes (DEGs) that were conserved across data sets were identified with FindConservedMarkers.

The Slingshot (2.6.0) and SingleCellExperiment (1.20.1) R packages were used to characterize global structure and predict lineages on the basis of cluster relationships. Slingshot performed trajectory inference with the dimensionality reduction produced by principal component analysis (PCA) and a set of cluster labels. To perform clonotype analysis, the filtered_contig_annotations.csv output from 10X Genomics Cell Ranger was loaded from each VDJ alignment folder to generate the data set used in scRepertoire (version 1.7.0). Extraneous prefixes of cell barcodes were removed with the stripBarcode function. A single list object of TCR genes and CDR3 sequences by cell barcode was combined with the integrated CD4^+^ T cell Seurat object using the “combineExpression” function. The different clonotypes frequencies were projected onto the Seurat object’s UMAP. CellChat R package (version 0.5.5) was used to make inferences about potential cell-cell interactions, as previously described (58).

### Statistics.

Statistical analysis was performed using GraphPad Prism 9 (GraphPad Software) or R for scRNA-Seq analysis. Unpaired 2-tailed *t* tests were used to compare 2 groups, whereas paired 2-tailed *t* tests were used for matched samples. Mann-Whitney *U* tests were used to compare 2 groups with nonparametric distribution. One-way ANOVA with Bonferroni’s post test was used for the comparison of multiple groups. Data with more than 1 variable was compared using a 2-way ANOVA followed by Bonferroni’s multiple-comparison test. For neuropathy incidence curves, a log-rank (Mantel-Cox) test was used. Bonferroni’s adjusted *P* values were reported for DEGs and Benjamini-Hochberg adjusted *P* values were used in pathway analysis. FCs were calculated as (B – A)/A. An adjusted *P* value of less than 0.05 was considered significant. Bar graph and dot plot data are presented as the mean ± SEM.

### Study approval.

All experiments with mice were approved by the UCLA Animal Research Committee.

### Data availability.

The data sets for scRNA-Seq and scTCR-Seq generated during the current study are available in the GEO repository (GEO GSE252646 and GSE252647; https://www.ncbi.nlm.nih.gov/geo/). Values for all data points in the graphs are reported in the Supporting Data Values file.

## Author contributions

MS, JKW, MGL, and MAS designed research studies. MS, MFB, and MM conducted experiments and acquired data. MS, MFB, ECM, SZ, HCC, MM, and WH analyzed data. JKW and SZ provided reagents. MS, MFB, ECM, MM, and MAS wrote the manuscript.

## Supplementary Material

Supplemental data

Supplemental table 1

Supporting data values

## Version 1 08/01/2024

Electronic publication
