# Supplementary material for: A pathologically expanded, clonal lineage of IL-21–producing CD4+ T cells drives inflammatory neuropathy
Source: J Clin Invest. 2024 Jun 11;134(15):e178602. doi: 10.1172/JCI178602 (PMC11290969; doi:10.1172/JCI178602)
Supplement: Supplemental data [file jci-134-178602-s133.pdf]

Supplemental Figures

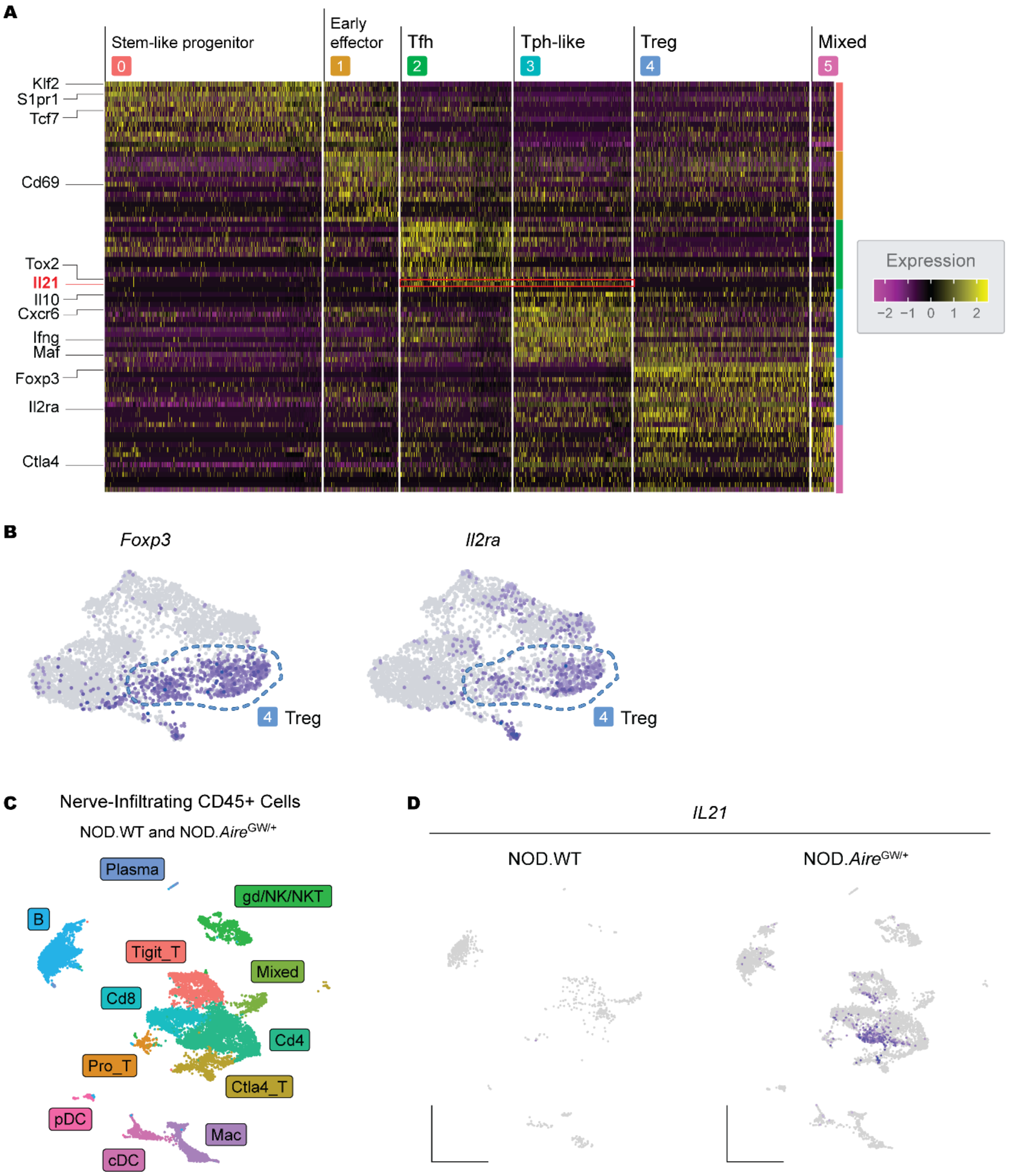

Supp. Fig. 1. (A) Heatmap showing top 14 DEGs for each cluster with key identifying genes bolded in left. **(B)** Feature plots of CD4+ T cells showing expression of regulatory T cell (Treg) genes (*Foxp3*, *Il2ra).* **(C)** Published UMAP (GSE 180498) of CD45+ T cells integrated from B6.WT, NOD.WT or NOD.Aire$^{GW/+}$ sciatic nerves. **(D)** Feature plots of CD45+ T cells showing expression of *Il21,* split by genotype (NOD.WT and NOD.Aire$^{GW/+}$)*.*

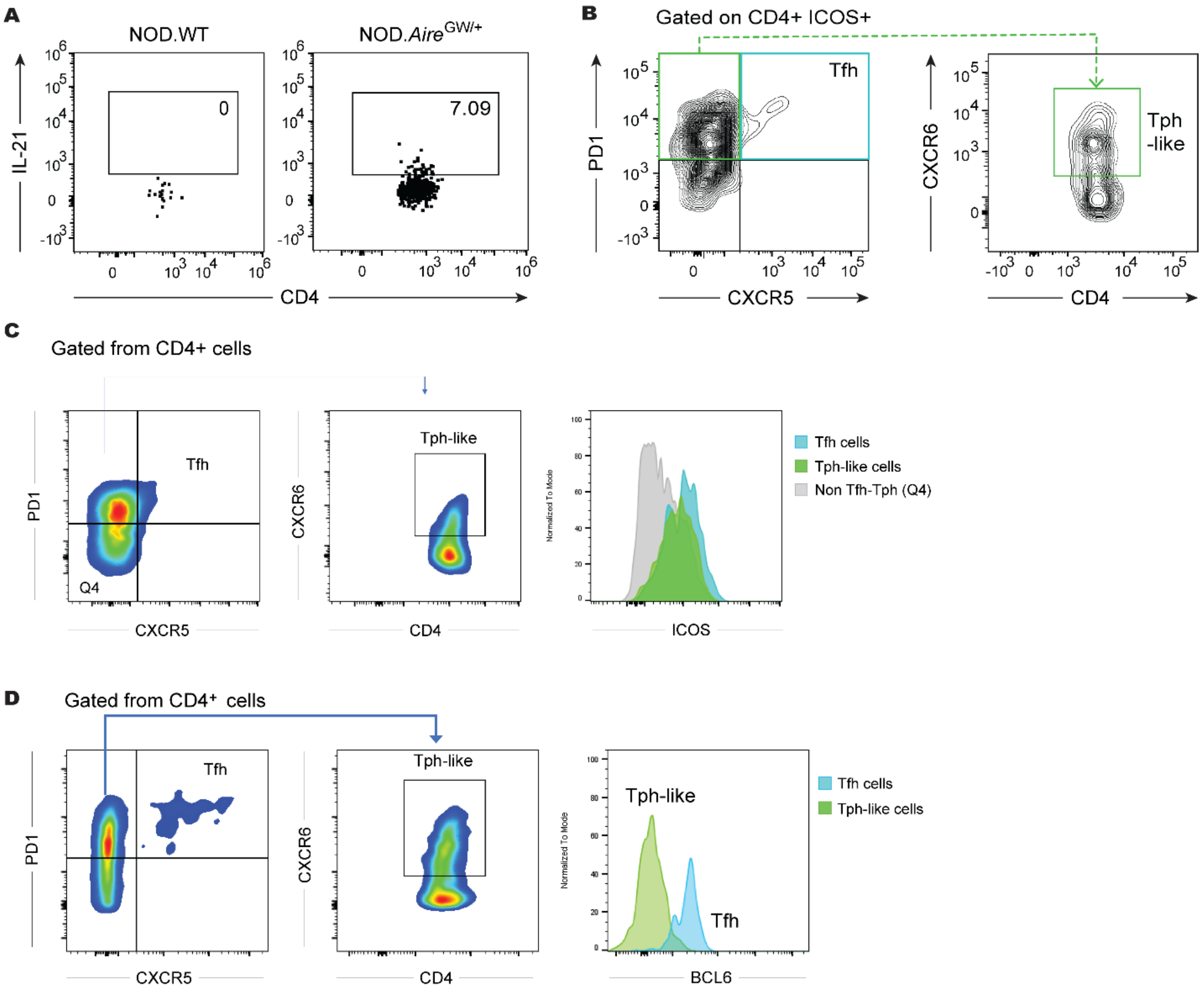

Supp. Fig. 2. IL-21 producing Tph-like cells in sciatic nerves of neuropathic NOD.Aire$^{GW/+}$ mice. (A) Representative flow cytometry plot of intracellular IL-21 staining of peripheral nerve CD4+ T cells from NOD.WT vs. neuropathic NOD.Aire$^{GW/+}$ sciatic nerves. **(B)** Gating strategy for flow cytometric analysis of Tfh (CD4+ ICOS+ PD1+ CXCR5+) and Tph-like cells (CD4+ ICOS+ PD1+ CXCR5- CXCR6+). **(C-D)** Representative flow cytometry graph of ICOS (C) and BCL6 (D) expression in Tfh and Tph-like cells infiltrating the peripheral nerve.

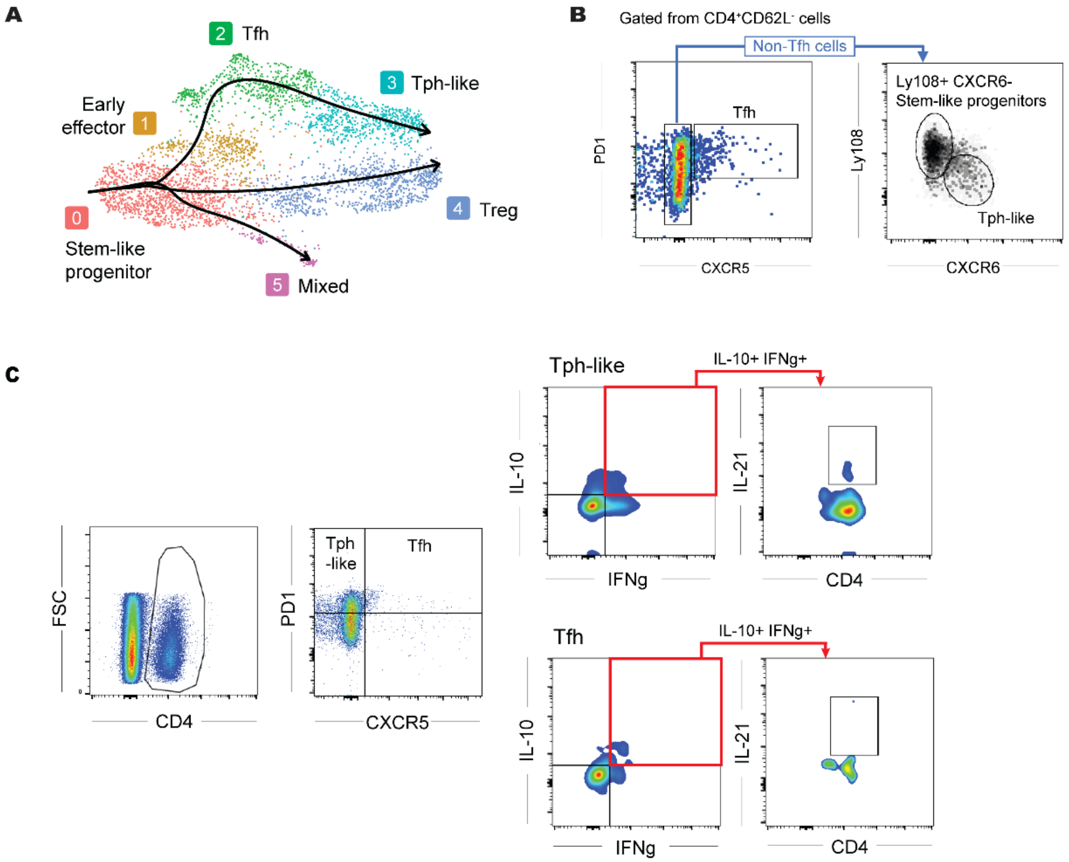

Supp. Fig. 3. Stem like progenitor cells, Tfh, and Tph-like cells in the peripheral nerves. (A) UMAP plot of CD4+ T cells with overlaid Slingshot pseudotime trajectories. **(B)** Flow cytometry plots for Tfh (PD1+ CXCR5+), Tph (CXCR6+ among non-Tfh), and stem-like progenitor CD4+ (Ly108+ among non-Tfh) cells in the peripheral nerves of a neuropathic mouse. **(C)** Representative flow cytometry graphs of CD4+ triple cytokine positive cells (IL-10+ IFN-γ+ IL-21+) among Tfh and Tph-like cells in the peripheral nerves of neuropathic mice.

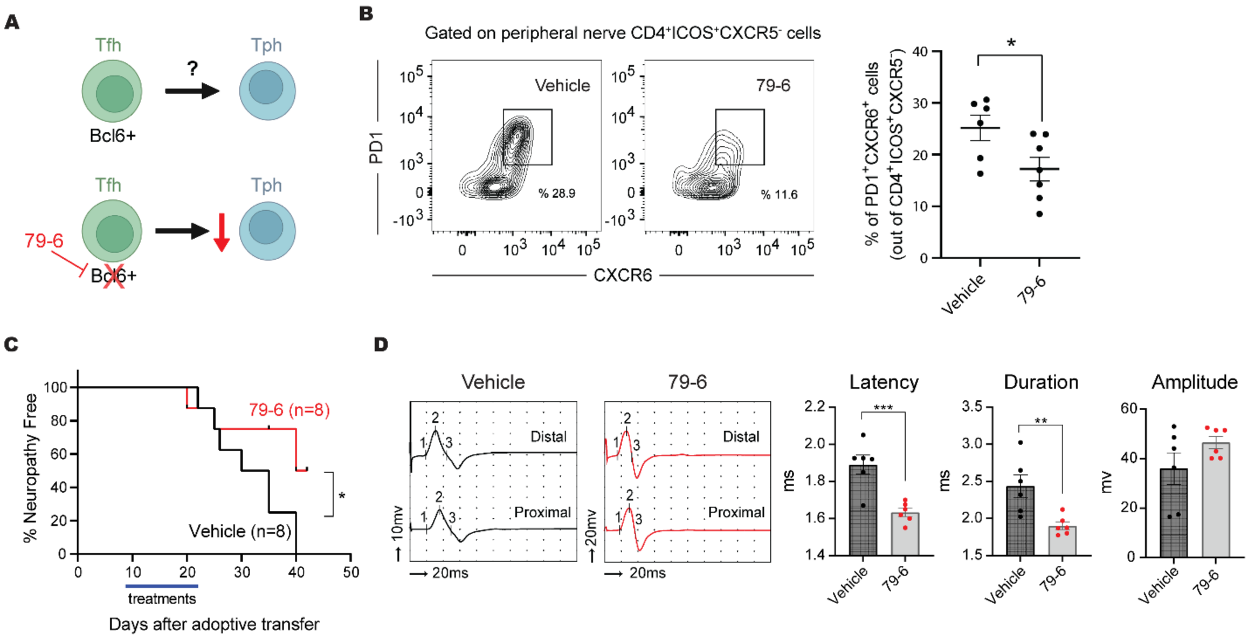

Supp. Fig. 4. BCL6 inhibition decreases Tph-like cells in the peripheral nerves and ameliorates neuropathy. (A) A model of how Tfh (BCL6+) cell differentiation to Tph-like (BLIMP1+) cells can be assessed by inhibiting BCL6 with 79-6. **(B)** Quantification of Tph-like cells in the peripheral nerves of neuropathic mice following adoptive transfer in the experimental groups (n=6, unpaired t-test). **(C)** Neuropathy incidence compared between experimental groups (n=8, Mantel-Cox test). **(D)** The compound muscle action potential parameters compared between 79-6 and vehicle treated groups (n=6, unpaired t-test).

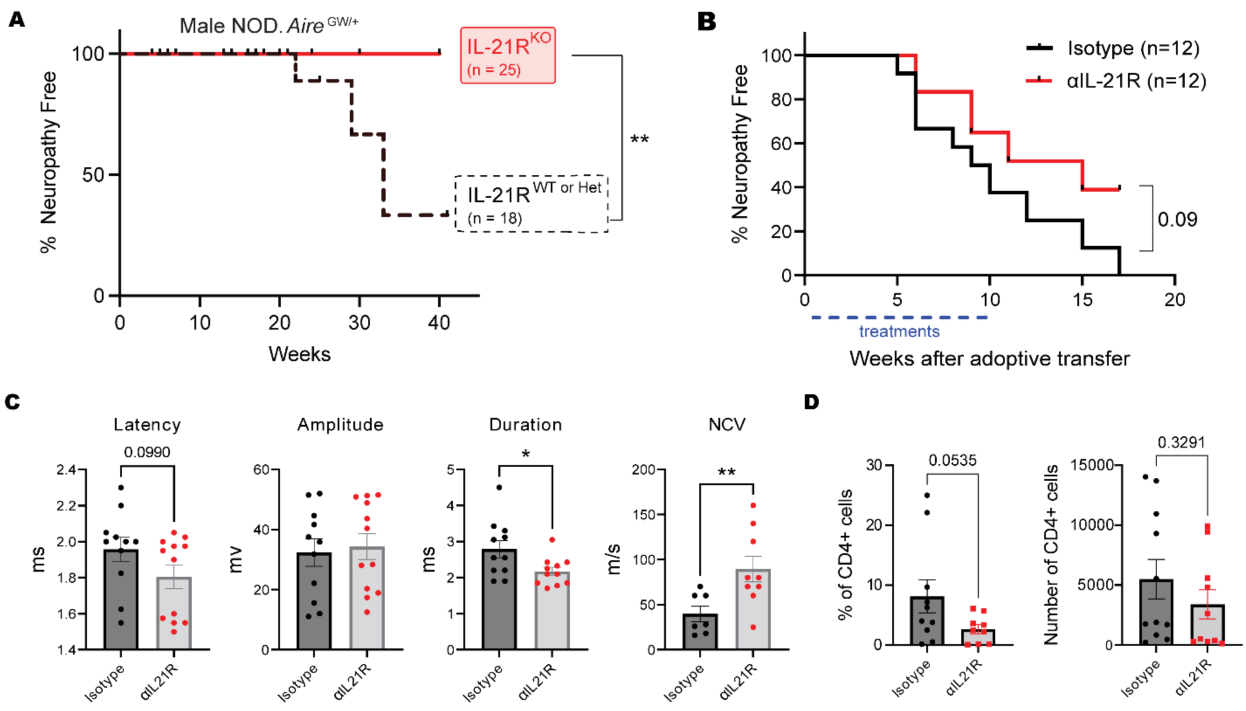

Supp. Fig. 5. IL-21R inhibition decreases the neuropathy incidence. (A) Neuropathy free incidence curve for male NOD.Aire$^{GW/+}$ IL21R^{KO} vs. NOD.Aire$^{GW/+}$ IL21R^{Het} and NOD.Aire$^{GW/+}$ IL21R^{WT} mice. Mantel-Cox test; **p<0.01. **(B)** The neuropathy incidence curve for anti-IL-21R or Isotype control treatment in an adoptive transfer model. Mantel Cox test. **(C)** Comparison of latency, amplitude, duration, and nerve conduction velocity (NCV) parameters from compound muscle action potentials between treatment groups (n=7-12, unpaired t-test). **(D)** The frequency and number of CD4+ cells in the peripheral nerves compared between groups (n=10-11, unpaired t-test).

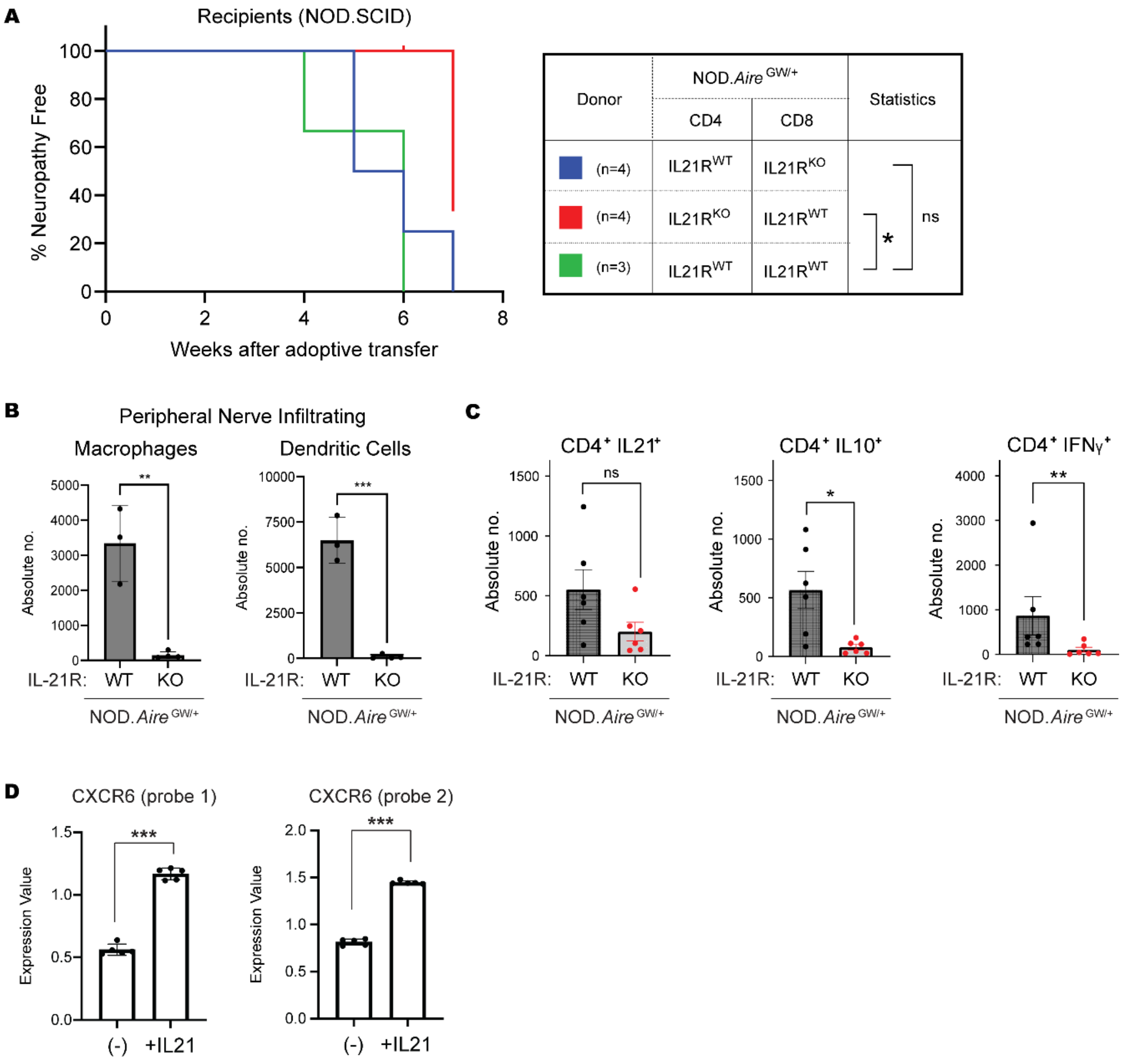

Supp. Fig. 6. IL-21 signaling modifies T cells and myeloid cells. (**A**) Neuropathy incidence curve of CD4+ and CD8+ cells from either NOD.Aire$^{GW/+}$ IL21R^{WT} or NOD.Aire$^{GW/+}$ IL21R^{KO} mice (150-500k cells) adoptively transferred into NOD.SCID recipients (n=3-4, Mantel Cox test). (**B**) The number of macrophages and dendritic cells in the peripheral nerves of the NOD.Aire$^{GW/+}$ IL21R^{WT} and NOD.Aire$^{GW/+}$ IL21R^{KO} mice (n=3, unpaired t-test). **(C)** Flow cytometric analysis of intracellular IL-21, IL-10, and IFN-γ staining of CD4+ T cells in peripheral nerves of Aire$^{GW/+}$

IL21R^{WT} vs. NOD.Aire$^{GW/+}$ IL21R^{KO} mice. **D)** CXCR6 expression levels after IL-21 stimulation of mouse CD4+ T cells at 24 hours. Data is plotted from Affymetrix gene expression data (GSE19198). Data are shown for the two microarray probes that map to CXCR6 [probe IDs 1425832_a_at (probe 1) and 1422812_at (probe 2)]. ***p<0.0001, n=5 per group. Student's t test.

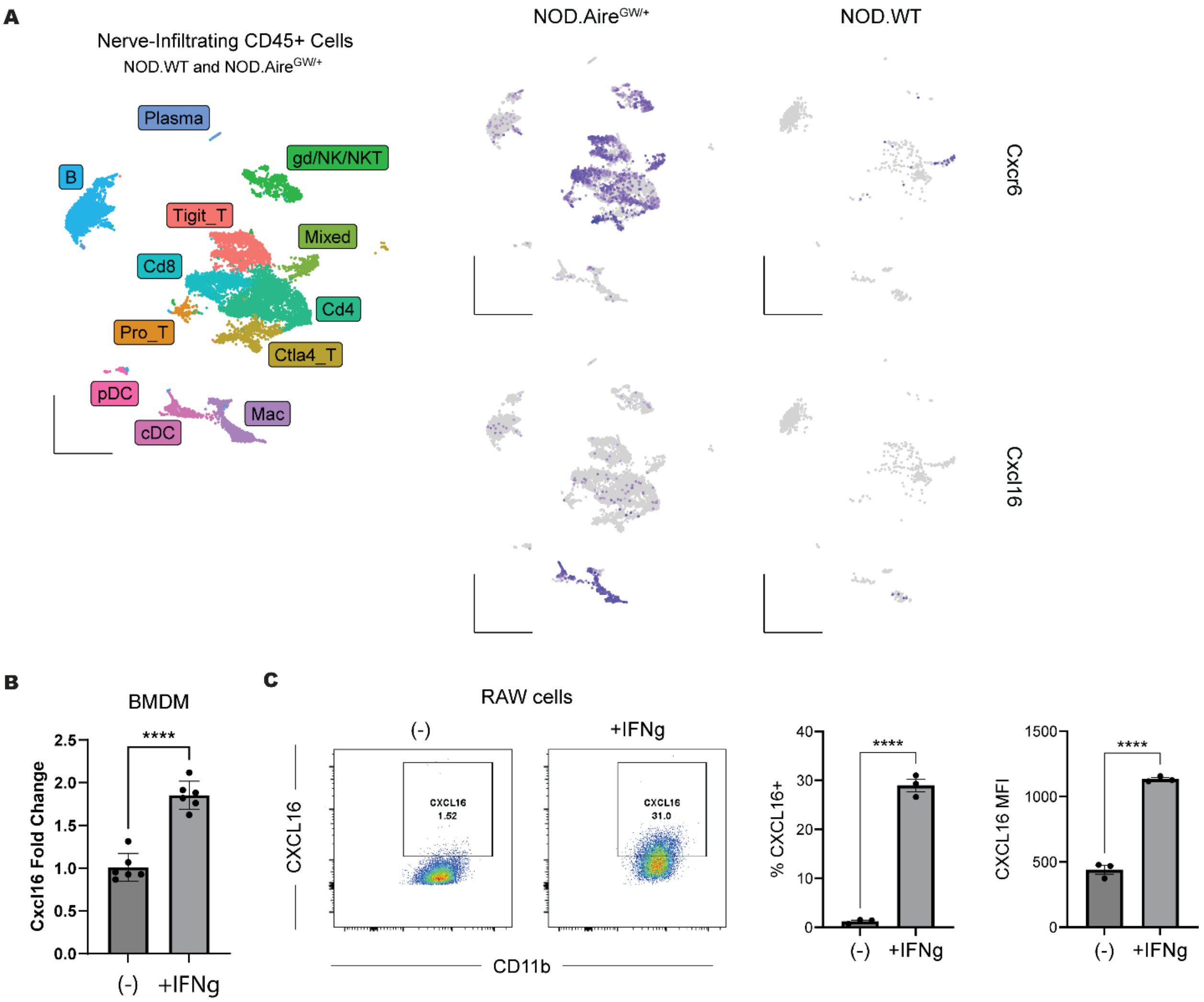

Supp. Fig. 7. CXCR6 and CXCL16 expression is upregulated in neuropathic NOD.AireGW/+ peripheral nerves. (A) Feature plots of CD45+ T cells showing expression of *Cxcr6 and Cxcl16,* split by genotype (NOD.WT and NOD.Aire$^{GW/+}$). UMAP (left) is from Supp. Fig. 1C. **(B)** Bone marrow derived macrophages (BMDM) from mice were left untreated (-) or treated with IFN-γ (40 ng/mL) for 8hrs. CXCL16 expression quantified by qRT-PCR (n=6, unpaired t-test). **(C)** CXCL16 expression quantified by flow cytometry in RAW cells, following IFN-γ (50 ng/mL) stimulation (n=3, unpaired t-test).
