# Supplementary material for: A pathologically expanded, clonal lineage of IL-21–producing CD4+ T cells drives inflammatory neuropathy
Source: J Clin Invest. 2024 Jun 11;134(15):e178602. doi: 10.1172/JCI178602 (PMC11290969; doi:10.1172/JCI178602)
Supplement: Supplemental table 1 [file jci-134-178602-s134.pdf]

Supplementary Table 1

Reactivity	Host	Antigen	Fluorophore	Clone	Supplier/Catalog Number
mouse	Rat	CD45	APC-eFluor 780	30-F11	Invitrogen/47-0451-82
mouse	Rat	CD4	Pacific blue	GK1.5	BioLegend/100428
mouse	Rat	CD4	BV605	RM4-5	BioLegend/100547
mouse	Rat	CD8	BV711	53-6.7	BioLegend/100747
human/mouse	Rat	CD45R/B220	PECy7	RA3-6B2	Invitrogen/25-0452-82
mouse	Rat	CXCR6	FITC	SA051D1	BioLegend/151108
mouse	Rat	CXCR5	PECy7	L138D7	BioLegend/145516
mouse	Rat	CXCR5	Biotin	2G8 (RUO)	BD Bioscience/551960
human/mouse/rat	Armenian Hamester	CD278(ICOS)	APC	C398.4A	BioLegend/313509
human/mouse/rat	Armenian Hamester	CD278(ICOS)	PECy7	C398.4A	BioLegend/313520
mouse	Armenian hamster	PD-1	PE	J43	Invitrogen/12-9985-82
mouse	Armenian hamster	PD-1	Super Bright 436	J43	Invitrogen/62-9985-82
mouse	Rat	IL-10	FITC	JES5-16E3	BioLegend/505006
mouse	Rat	IL-21	PE	mhalx21	Invitrogen/12-7213-82
mouse	Rat	IFN-γ	APC	XMG1.2	BioLegend/505810
mouse/human	Rat	BCl-6	APC	7D1	BioLegend/358505
		CXCL16	PE		
		CD11b			
mouse	Rabbit	CXCL16	unconjugated	-	Bioss/ BS-1441R
mouse	Rabbit	IL-21	unconjugated	-	Invitrogen/ PA5-115407
Rabbit	Goat	-	AF488	-	Invitrogen/A-11008
Streptavidin (APC)					Bioscience/ 17-4317-82
